# Chloroform in Paroxysmal Typhoid Fever

**Published:** 1857-12

**Authors:** F. W. White


ARTICLE V.

### CHLOROFORM IN PAROXYSMAL TYPHOID FEVER.

BY F. W. WHITE, M.D.

In the beginning of November, I was called to see Mrs. B——. She was a native of New England, and only for a few weeks previously a resident of Chicago, having moved west in the month of September. She was 25 years of age, and of good constitution.

From the history of the case as given by herself and friends, I elicited the following information: She had complained for nearly a week prior to this of slight pain in the head, back and limbs; lassitude, restlessness and anxiety; loss of appetite, uneasiness in the epigastrium and nausea. There had first been a slight chill, which was followed by remissions and exacerbations of fever, with a sense of chilliness during every day.

At five o'clock P. M. during this my first visit, the following symptoms were manifest: The surface of the body was hot and dry, the face flushed, lips of a purple hue, eyes dull and heavy, and the countenance anxious and haggard. The pulse was frequent, but not quick or sharp, soft, easily compressed and somewhat irregular, and about 110 in a minute. There was pain in the head, back and limbs. The tongue was covered with a thin white fur, nausea, pain in the epigastrium, and bowels constipated. There had been a remission of the febrile excitement during the morning, but not an entire intermission. The urine was scanty, and of a deep red color.

Taking the history of the case and the symptoms apparent at this time, I diagnosed a simple remittent or bilious fever, and prescribed the following:

| | | |
|---|---|---|
| ℞ | Sub. mur. hydr., grs. | xjj. |
| | Pulv. Doveri. | ʒj. |
| | Sulph. quiniæ. | ℈j. |

M. Fiat pulv. no. vj. Of these powders I directed one to be taken every four hours, alternated by one-half teaspoonful of spts. nit. dulcis. during the remission, the spts. nit. dulcis. to be continued during the paroxysm of fever, at intervals of two hours.

*Second day, 3 o'clock p. m.*—Passed a restless night, being quite nervous and confused, which was followed early in the morning with decided remission and sense of chilliness. These symptoms passed away before mid-day, and I found her in the afternoon with a full soft pulse, about 110 in a minute, throbbing headache, pain in the back and limbs, and a sense of general weariness. The skin was hot and dry, face much flushed ; lips of a deeper purple hue ; countenance anxious, and there was slight delirium ; tongue heavily coated with a dirty white fur ; no appetite ; great thirst ; nausea. I directed a laxative to be administered, and the powders to be continued as during the previous day. These powders were designed to open the secretions, and break up, if possible, the apparent periodical character and return of the fever. To meet the nervous symptoms and quiet the generally irritable condition of the system, I prescribed fifteen drops of chloroform, to be given in each dose of the spts. nit. dulcis., this to be taken with half a wineglass of sweetened water, and continued during the night.

*Third day.*—Patient rested well during the night ; nervous symptoms somewhat less intense ; remission of fever as on previous day, but not of so long duration, nor of so marked a character. The bowels moved excessively after the administration of the laxative.

*Three o'clock p. m.*—The exacerbation of the fever continued unchanged in any perceptible degree ; restlessness ; insomnia ; eyes injected, heavy and dull ; pain in the head and back ; countenance much flushed ; lips deeply purple ; pulse 110 in a minute, soft, small, feeble and easily compressed ; tongue dry, heavily coated and red at the edges ; bowels tender, loose, with watery discharges ; pain in the right iliac region on pressure ; tympanitic ; urine scanty, high colored and offensive. She was apparently much depressed. The nervous symptoms being modified in a considerable degree, and considering the chloroform too irritating to the mucous membrane, I ordered it to be discontinued, and finding the quinine did not exert the desired influence in arresting the paroxysmal character of the disease, but rather appearing to increase the exacerbations, I had it also discontinued.

Finding, now, the well marked symptoms of typhoid fever, with the exception that there was a remission of a peculiar and unmistakable character, with daily exacerbations or paroxysms of fever, I diagnosed a typhoid fever of the remittent type or a paroxysmal typhoid fever, as more properly denominated by Prof. N. S. Davis.

The patient being much depressed and prostrate, I prescribed as follows :

| | | | |
|---|---|---|---|
| ℞ | Sulph. morphiæ, | gr. | j. |
| | Sulph. quiniæ, | grs. | v. |
| | Mur. sodæ, | ℈ | iv. |

M. Ft. pulv. no. v. One to be administered every three hours, and a Dover's powder to be given at night. By this means I designed to fulfil two indications,

1st. To allay the irritability of the stomach and bowels.

2d. To increase the tonicity of the system.

*Fourth day*, 8 *o'clock a.m.*—Found the patient with the usual remission of fever, but not so complete nor of so long duration. Nervous symptoms somewhat more unfavorable; other symptoms more favorable than on the previous day. Treatment continued.

*Three o'clock p. m.*—Found the patient with an increased paroxysm of fever; nervous symptoms all intensified; pulse more frequent and feeble; skin burning hot, arid and crispy; tongue more dry, brown and cracked; pain in the abdomen; bowels tympanitic and more irritable, with frequent discharges of a watery character; urine scanty, high colored and offensive. The expression of the countenance was vacant, more anxious, of a deeper, dusky hue; eyes injected. Delirium of a low muttering kind. Having witnessed the good effects from the administration of chloroform in cases of typhoid fever, where the pulse was soft and easily compressed, and where the nervous symptoms are more predominant (in my own practice, and that also of Prof. N. S. Davis), I determined to rely on it mainly in this case.

Chloroform being almost entirely insoluble in water, and when in combination with sweet spts. nitre and water is precipitated, and passing over the mucous membrane of the throat to that of the stomach, in the undiluted form produces a considerable inflamation, almost amounting to acute gastritis. Having witnessed this result in several cases, I devised the following emulsion :

℞ Pulv. acaciæ,

Pulv. acchari. albi. aa ʒjjss.

Spts. nit. dulcis.

Chloroform aa ʒjss.

Aquæ destil. ℥jss.

Ft. emulsio. Of this I directed two teaspoonfuls every four hours, alternated with one of the following powders:

℞ Pulv. oppii. grs. vi.
Sulph. quiniæ grs. vi.
Sacch. albi. ʒss.

M. ft. pulv. no. vi. I designed, in this instance, to increase the tonicity of the system, allay the nervous susceptibility, and quiet the intestinal irritation, and thus causing the pulse to become slower and more full, the vital forces would be increased.

*Fifth day.*—Saw the patient at 10 o'clock a. m. A slight remission during the morning; pulse 100 in a minute; soft, and not so small as before; tongue brown, cracked and dry; skin hot and dry; delirium not so great—not so restless. Treatment continued.

*Six o'clock p. m.*—Pulse 100 in a minute, more full than in the morning, soft and easily compressed; less anxiety manifest; slight delirium; face less dusky; tongue brown and more cracked than before; complains of fauces being sore; bowels more quiet during the day; less tympanitic. Treatment continued.

*Sixth day, 3 o'clock p. m.*—The patient rested better during the night; had very little fever during the morning. Pulse is now 90 in a minute, more full and regular; tongue cleaning off some, through the centre, and less dry; bowels less troublesome; no delirium; skin not so dry; patient in every way improved. Continued the treatment at longer periods.

*Seventh day.*—Good night's rest; all the symptoms much improved. Directed the emulsion to be used every six hours, and the powders to be substituted by one teaspoonful of the following, between each dose of the chloroform:

℞ Magnesia sulphat ʒjj.

Tinct. oppii. ʒjj.

Aromat. sulph. acid dilut. ʒj.

Aquæ ℥jj.

M. To be taken in a little sweetened water, and to be continued as long as the bowels should remain too loose.

These were continued until the evening of the eighth day, when she was put upon tonics and simple nutritious diet. She had strong beef tea, highly salted, during the course of the fever, as much as she could be induced to take. She went on convalescing until entirely well.

I administered, in this case, quinine in small doses most of the time, after failing to break up the paroxysms of fever, not for the purpose of cutting short the disease, but to sustain the system. I assign the favorable termination of the fever to the use of the chloroform emulsion for the most part; for it will be noticed when the chloroform was discontinued and the quinine substituted and combined in the salt mixture, the symptoms all became more unfavorable.

The "modus operandi" of the chloroform in the peculiar pathological conditions existent in such cases is not fully known. By inhalation we know that it is first stimulant, increasing the susceptibility of the system, exciting and quickening the pulse, and afterward altering its character to a slow, full, but not hard pulse.

By actual experiment upon myself, taken in doses of fifteen drops every half hour, the pulse rose first, and in an hour fell below the usual number of pulsations.

The use of chloroform is therefore based on experience for the greater part. The patient, in the present case, was sick about twelve or thirteen days in all, and about eight after treatment was commenced.

